# Fecundity compensation and tolerance to a sterilizing pathogen in *Daphnia*

**DOI:** 10.1111/j.1420-9101.2012.02579.x

**Published:** 2012-08-01

**Authors:** P F Vale, T J Little

**Affiliations:** * Centre d’Ecologie Fonctionnelle et Evolutive (CEFE-CNRS), UMR 5175Montpellier, France; † Institute of Evolutionary Biology, School of Biological Sciences, University of EdinburghEdinburgh, UK

**Keywords:** *Daphnia*, fecundity compensation, invertebrate, *Pasteuria*, resistance, sterilization, tolerance

## Abstract

Hosts are armed with several lines of defence in the battle against parasites: they may prevent the establishment of infection, reduce parasite growth once infected or persevere through mechanisms that reduce the damage caused by infection, called tolerance. Studies on tolerance in animals have focused on mortality, and sterility tolerance has not been investigated experimentally. Here, we tested for genetic variation in the multiple steps of defence when the invertebrate *Daphnia magna* is infected with the sterilizing bacterial pathogen *Pasteuria ramosa*: anti-infection resistance, anti-growth resistance and the ability to tolerate sterilization once infected. When exposed to nine doses of a genetically diverse pathogen inoculum, six host genotypes varied in their average susceptibility to infection and in their parasite loads once infected. How host fecundity changed with increasing parasite loads did not vary between genotypes, indicating that there was no genetic variation for this measure of fecundity tolerance. However, genotypes differed in their level of fecundity compensation under infection, and we discuss how, by increasing host fitness without targeting parasite densities, fecundity compensation is consistent with the functional definition of tolerance. Such infection-induced life-history shifts are not traditionally considered to be part of the immune response, but may crucially reduce harm (in terms of fitness loss) caused by disease, and are a distinct source of selection on pathogens.

## Introduction

Amidst the widespread threat of parasitism, hosts persist with the help of several lines of defence (Frank, 2002). The first line of defence is achieved through resistance mechanisms that prevent infections from establishing (anti-infection resistance). There is a great diversity of mechanisms that mediate anti-infection resistance, from nonspecific physical and chemical barriers (Canny *et al*., 2002; Corteel *et al*., 2009) to other more specific mechanisms of entry based on effector–receptor molecule recognition (e.g. Bergelson *et al*., 2001; Frank, 2002; Duneau *et al*., 2011). If breached, a second line of chemical and cellular responses target pathogen within-host growth, leading to lower infection loads (anti-growth resistance) (Frank, 2002; Kurtz, 2005). Genetic variation in host resistance traits is widespread, and its maintenance is thought to arise due to a combination of frequency-dependent selection (Hamilton, 1993; Lambrechts *et al*., 2006), costs of resistance (Sheldon & Verhulst, 1996; Moret & Schmid-Hempel, 2000) and variable infection prevalence (Boots *et al*., 2009; Laine *et al*., 2011). Both anti-infection and anti-growth resistance can independently follow different models of infection genetics (Agrawal & Lively, 2002) with substantial implications for co-evolution (Agrawal & Lively, 2003; Fenton *et al*., 2012).

If parasites evade both anti-infection and anti-growth defences, it is still possible for hosts to reduce the harm caused during infection through tolerance mechanisms that maintain host health and fitness without necessarily reducing parasite densities (Read *et al*., 2008; Schneider & Ayres, 2008; Råberg *et al*., 2009). For example, mechanisms that interfere with infection-derived toxins (Feingold *et al*., 1995; Pamplona *et al*., 2007; Rasko & Sperandio, 2010) result in tolerance because they reduce the severity of disease that arises from infection, without directly reducing the total density of pathogens. Other mechanisms, such as wound repair during infection (Reece *et al*., 2006; Ayres & Schneider, 2008), or those that reduce immunopathology (Graham *et al*., 2005), have also been proposed as potential promoters of tolerance to infection, because parasite growth is not directly targeted but the net result is a fitter host.

The mechanisms maintaining variation in tolerance are less clear than for other defence traits, and it may be helpful to distinguish between the ways in which tolerance promotes host fitness. Hosts may tolerate infection by living longer than non-tolerant hosts for a given infection load (*mortality tolerance*). By evolving tolerance to high parasite burdens, hosts become potential transmission hotspots, ultimately leading to higher infection prevalence (Miller *et al*., 2006). This creates a positive feedback where high tolerance benefits both the host (because virulence is relatively lower) and the parasite (because prevalence is relatively higher), which suggests that alleles underlying mortality tolerance will become fixed (Roy & Kirchner, 2000). An alternative way of tolerating infection is by maintaining reproduction during infection (*sterility tolerance*) (Best *et al*., 2008, 2010). Tolerating infection by maintaining reproduction would clearly benefit the host, but the benefit for the parasite is less clear. Increased host reproduction might reduce the infectious period (if reproduction obeys a trade-off with survival), it might divert host resources that may ultimately lead to lower pathogen loads [e.g. Ebert *et al*. (2004)], but it could also increase the number of susceptible hosts in the population. Theoretical work suggests that maintaining polymorphism in sterility tolerance is more likely than mortality tolerance, and this will depend largely on the prevalence of infection and the cost of tolerating infection (Best *et al*., 2008).

Despite these important differences between mortality and fecundity tolerance, experimental investigation of tolerance in animals has largely focused on host survival (Corby-harris *et al*., 2007; Råberg *et al*., 2007; Ayres & Schneider; 2011; Lefèvre *et al*., 2011), rather than damage control through increased fecundity (sterility tolerance). Here, we tested for genetic variation at several steps that comprise the defence against infection in the invertebrate *Daphnia magna* to the sterilizing pathogen *Pasteuria ramosa*: anti-infection resistance, anti-growth resistance and, especially, fecundity tolerance. Testing for tolerance requires a measure of how host fitness changes with increasing pathogen load (Simms, 2000; Råberg *et al*., 2009; Little *et al*., 2010). As *P. ramosa* is a sterilizing parasite, we took the level of fecundity under infection as an indicator of host fitness [see also Best *et al*. (2010)]. We followed an infection protocol that measured these stages of defence across a large range of infection doses of a genetically diverse pathogen inoculum (Ben-Ami *et al*., 2010; Lefèvre *et al*., 2011), the goal being to assess the average host resistance and tolerance under a range of infection conditions that are similar to what hosts would naturally experience.

## Materials and methods

### The model system

*Daphnia magna* are planktonic crustaceans found in most temperate freshwater ponds and lakes. *Daphnia* used in this study were hatched from ephippia (*Daphnia* resting eggs, produced through sexual reproduction) isolated from a thin, uppermost layer of sediment collected from the Kaimes pond near Leitholm, in the Scottish Borders (2°20.43 ′W, 55°42.15 ′N). Each individual female hatchling is a genetically unique clone resulting from sexual reproduction and was propagated in a state of clonal reproduction since their hatching in 2007. In this study, we used six randomly chosen clones labelled KA2, KA7, KA18, KA47, KA48 and KA81. The parasite *P. ramosa* is a gram-positive, spore-forming, obligate bacterial parasite of *D. magna*. Infection occurs during filtration feeding by ingestion of parasite spores, and leads to host sterilization and premature death. Infected hosts are visible by naked eye due to their red coloration and absence of eggs in the brood chamber. Sterilization is not reversible and usually occurs after hosts have produced one or two clutches (although complete sterilization is common). The parasite isolates used in the experiment originated from the same Kaimes population. A general spore suspension was used, made by macerating and combining several *Daphnia* infected with *P. ramosa* from this population.

### Infection procedure

Hosts were exposed after a period of acclimation. Twelve independent replicate jars (60 mL) containing three *Daphnia* per jar were maintained with artificial pond medium (Klüttgen *et al*., 1994) for three generations in identical food (*Chlorella vulgari*s microalgae; 1 abs per daphnia per day), temperature (20 °C) and light conditions (12:12-h light/dark). Hosts were exposed to parasite transmission spores at nine doses [0, 25, 50, 100, 125, 150, 175, 200 and 400 (×10^3^) spores]. We prepared several aliquots in 1.5-mL Eppendorf tubes, each containing 1 mL of the appropriate dilution, so that adding 100 μL to each host jar would achieve the desired dose. We prepared one tube per dose per infection replicate, which allowed the parasite suspension to be independent at the level of host replicates (e.g. replicate one of each six host genotypes received spores from parasite tube 1 and so on). Infection was carried out in 24-well cell culture plates containing 5 mL of synthetic pond medium and 100 μL of the desired spore suspension, with one *Daphnia* per well. Exposure was done overnight, lasting 18 h. Given estimates of *Daphnia* spp. filtration rate between 2 and 4 mL per individual per hour [See Lampert in Peters & De Bernardi (1987)], this exposure protocol allows the total volume of the well, including parasite spores, to be filtered and ingested between 7 and 14 times.

### Observation period

Following exposure, each *Daphnia* was transferred to a small glass jar containing 60 mL of artificial pond medium and fed one absorbance (the optical absorbance of 650-nm white light by the *Chlorella* culture, with 1.0 absorbance being equivalent to approximately 5 × 10^6^ algal cells) per *Daphnia* per day of *C. vulgaris*, a green microalgae cultured in chemostats with Chu B medium. The observation period lasted 40 days, and during this period, we recorded signs of infection, counted the number of offspring produced in each clutch and monitored for death. We counted the number of *P. ramosa* transmission spores present on day 40 post-infection, when all infected *Daphnia* were individually placed in an Eppendorf tube and stored at −20 °C. We added 500 μL of sterile water to each tube and crushed *Daphnia* using a motorized Pellet Pestle. Spore counts were achieved by adding 50 μL of the thoroughly mixed spore suspension to 10 mL of CASYton isotonic solution and reading this dilution on a CASY® Cell Counter Model TT (Schärfe System GmbH, Reutlingen, Germany).

### Analysis

We analysed differences among genotypes in (i) anti-infection resistance; (ii) anti-growth resistance (infected *Daphnia* only); (iii) tolerance to pathogenesis, measured by the ability to sustain fecundity under infection. We exposed a total of 576 individual *Daphnia* from six genotypes to eight doses of *P. ramosa*, plus a total of 72 unexposed controls (12 replicates per treatment). Thirty-eight *Daphnia* died before infection status could be established and were removed from the analyses. Anti-infection resistance was analysed as the fraction of hosts that remained uninfected after exposure, using a generalized linear model with binomial errors. Anti-growth resistance was inferred from the parasite load in each infected *Daphnia* 40 days post-exposure, and this was analysed in a general linear model with normal errors (residuals deviated slightly from a normal distribution, but transforming the data did not improve the model fit nor change the results of the analysis). Both analyses included host genotype and dose as fixed effects, plus a quadratic term for dose to account for a nonlinear relationship between dose and the response variable. Models were reduced by removing the highest-order nonsignificant term until all remaining terms were significant. To analyse sterility tolerance, we asked whether host genotypes varied in the ability to maintain reproduction with increasing pathogen loads. For this, we determined whether these two traits co-varied differently among host genotypes (See Graham *et al*. 2010). We performed a separate multivariate analysis of variance (manova) for each host genotype with both ‘host fecundity’ and ‘parasite spore load’ as response variables and ‘inoculation dose’ as a fixed effect. From these analyses, we extracted the genotype-specific correlation coefficients between the two response variables. Negative correlations indicate a loss of fecundity with increasing parasite load, whereas correlations not different from zero suggest that host genotypes tolerate increasing parasite loads without suffering a reduction in fecundity. We tested whether genotype-specific correlations differed using Fisher’s Z transformation (Fisher, 1915), which transforms Pearson’s correlation coefficients (*r*_*i*_) into normally distributed *Z*_*i*_ variables, where

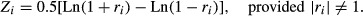


This allows the difference between bivariate correlations to be tested using the χ^2^ test statistic, where

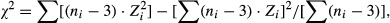

which compares the variability in correlations for the sample size *n*_*i*_, under the null hypothesis that all correlation coefficients are equal (Fisher, 1970). This analysis was carried out using the online analysis tool available at http://home.ubalt.edu/ntsbarsh/Business-stat/otherapplets/MultiCorr.htm (accessed December 2011).

We further considered that the amount of reproduction achieved by an infected host could be in part due to fecundity compensation, that is, when infected hosts increase early reproduction relative to uninfected hosts. To assess fecundity compensation, we compared the reproduction of an infected host before sterilization occurs, relative to the reproduction of an uninfected host during the same period. Given the variable time period before sterilization among hosts, we chose to compare the difference in the number of offspring produced in the first clutch between infected hosts and those that received zero spores [see also Chadwick & Little (2005)]. This was analysed using a general linear model, with host genotype, infection dose and their interaction as fixed effects, plus a quadratic term for dose to account for a nonlinear relationship between dose and the response variable. As mentioned earlier, models were reduced by removing the highest-order nonsignificant term until all remaining terms were significant.

## Results

### Host genotypes differ in resistance

**Figure 1:**
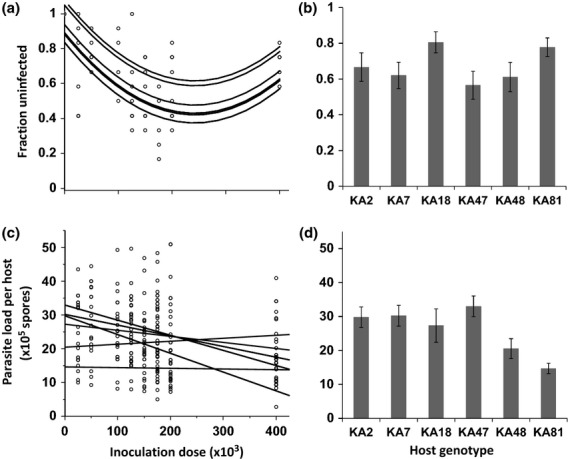
Resistance to infection and within-host growth. (a) Each line is the least-squares regression for the fraction of uninfected individuals for each host genotype plotted against inoculation dose, obtained from the best generalized linear model (*F*_7,46_ = 9.43, *P* < 0.0001, *R*^2^ = 0.59; see Table 1). (b) The mean fraction of uninfected (± standard errors), across all inoculation doses, for each host genotype. (c) Least-squares regressions for the number of parasite spores per infected host (parasite load), plotted against inoculation dose, for each host genotype, obtained from the best generalized linear model (*F*_11,191_ = 4.39, *P* < 0.0001, *R*^2^ = 0.20; see Table 1). (d) The mean parasite load (± standard errors), across all inoculation doses, for each host genotype.

The first line of defence against infection is to prevent parasites from gaining entry into the host. A total of 202 *Daphnia* (33%) developed infection across all infection doses. The proportion of hosts resisting infection varied with dose (Table 1) and with host genotype (Table 1, Fig. 1a,b). If the initial barrier to infection is breached, hosts are still able to reduce the burden of infection by reducing parasite loads. Host genotypes differed in their parasite loads measured on day 40 post-infection, and this burden varied according to the initial dose of parasite inoculum (Table 1; Fig. 1c,d).

**Table 1:** GLM analysis of the effects of host genotype and inoculation dose on different stages of defence

Source	d.f.	*F*	*P*
Proportion resistant (infectivity resistance)			
Host genotype	5	3.65	0.0073
Inoculation dose	1	46.11	< 0.0001
(Inoculation dose)^2^	1	35.55	< 0.0001
Parasite load (growth resistance)			
Host genotype	5	4.97	0.0003
Inoculation dose	1	12.16	0.0006
Host genotype × Inoculation dose	5	2.10	0.0673
Host fecundity			
Host genotype	5	2.43	0.0365
Inoculation dose	1	8.08	0.0049
Host genotype × Inoculation dose	5	2.57	0.028
Fecundity compensation			
Host genotype	5	24.15	< 0.0001
Inoculation dose	1	13.99	0.0002

d.f., degrees of freedom; *F*, *F*-ratio.

### Host genotypes do not differ in sterility tolerance

**Figure 2:**
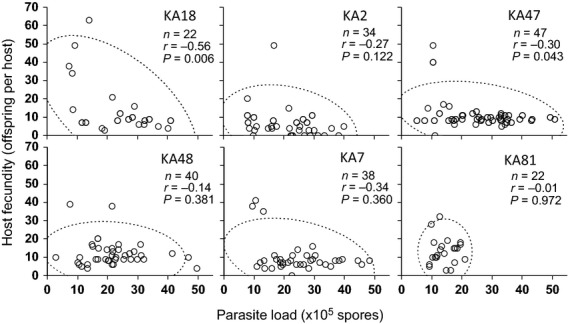
The correlation between host fecundity and parasite load. Measured on day 40 post-infection for each host genotype and for all inoculation doses. Negative correlations indicate a loss of fecundity with increasing parasite load, whereas correlations not different from zero suggest that host genotypes tolerate increasing parasite loads without suffering a reduction in fecundity. Note that while we present the individual correlation coefficients, a complete analysis revealed no significant difference in the correlation between host genotypes (see text for details). Ellipses are 95% confidence intervals. *r*, Pearson’s correlation coefficient; *n*, sample size.

If pathogens bypass both anti-infection and anti-growth resistance, sustained within-host growth will ultimately result in a reduction in host fitness, either due to exploitation of host resources that could otherwise be allocated to host growth and reproduction or due to damage caused to host tissues. However, hosts may still reduce this damage via tolerance mechanisms that improve fitness without targeting parasites directly. We analysed how host fecundity changed with increasing spore loads for each genotype and found that genotype-specific correlations were broadly not different from zero (Fig. 2). This result suggests that before sterilization is complete, hosts are able to maintain similar levels of fecundity across a range of parasite loads. Correlation coefficients did not differ significantly between the genotypes tested (χ^2^ = 0.74616, *P* = 0.739), indicating little variation in the ability to tolerate the reduction in fecundity during infection.

### Host genotypes differ in fecundity compensation

**Figure 3:**
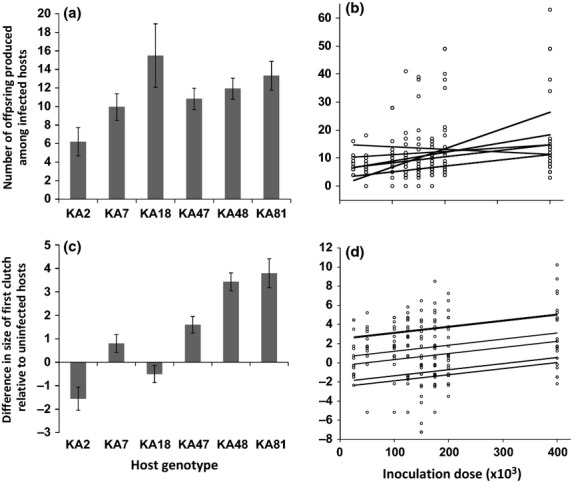
Fecundity compensation. (a) The mean number of offspring (± standard errors), across all inoculation doses, for each host genotype. (b) Least-squares regressions for the number of offspring per infected host, plotted against inoculation dose, for each host genotype, obtained from the best generalized linear model (*F*_11,190_ = 4.23, *P* < 0.0001, *R*^2^ = 0.20; see Table 1). (c) The mean level of fecundity compensation (± standard errors), measured as the difference in number of offspring of the first clutch had by infected individuals, relative to the first clutch of uninfected individuals of the same genotype. (d) Least-squares regressions for the level of fecundity compensation, plotted against inoculation dose, for each host genotype, obtained from the best generalized linear model (*F*_6,195_ = 922.07, *P* < 0.0001, *R*^2^ = 0.40; see Table 1).

Despite finding no difference in how fecundity co-varied with parasite loads, genotypes differed in their total fecundity under infection (Table 1, Fig. 3a). Therefore, when faced with infection by a sterilizing parasite, some hosts show greater fitness than others. We tested the possibility that this difference arose due to fecundity compensation, an increase in reproductive output in the early stages of infection. Two genotypes (KA2 and KA18) had on average smaller first clutches than their uninfected equivalents, whereas the remaining four genotypes had on average between 1 and 3.5 extra offspring in the first clutch compared to uninfected hosts of the same genotype (Fig. 3c). For all genotypes, fecundity compensation was higher with increasing inoculation doses (Table 1, Fig. 3d).

### Genetic correlations between multistep defences

We tested the genetic correlation (i.e. taking each of the mean value for each clone as a single data point) between the level of fecundity compensation and the two measures of resistance. Across all inoculation doses, we found no evidence for a trade-off between fecundity compensation and either anti-infection resistance (*r* = 0.004, *n* = 6, *P* = 0.99) or anti-growth resistance (*r* = −0.774, *n* = 6, *P* = 0.071). We further examined the phenotypic correlations within each clone, but these also showed no relationship between the degree of fecundity compensation and, for example, anti-growth resistance (largest *r*^2^ = 0.12, all tests not significant).

## Discussion

Infection is inherently a multistep process, and it is important to consider host defences at distinct stages of infection. Resistance mechanisms that target pathogens directly have been widely investigated, but it is becoming increasingly clear that alternative ways of improving host fitness, such as tolerance, also play an important role in reducing the harm caused during within-host growth. Genetic variation was present in several stages of defence when *D. magna* is infected with sterilizing pathogen *P. ramosa*. Apart from varying in their ability to limit infection at the stage of initial infection, and later varying in their parasite burdens, we also found genetic variation for fecundity compensation. Below, we discuss how fecundity compensation may be viewed as a tolerance mechanism, by increasing host fitness via mechanisms that do not act directly on reducing parasite densities. Exploring such defence mechanisms that are not traditionally considered immune responses (Parker *et al*., 2011), and yet may form an important part of reducing the harm caused by disease, will help elucidate the numerous and distinct sources of selection on pathogens, which in turn determine both epidemiological and evolutionary outcomes.

### Variation in fecundity tolerance

Theoretical models of resistance and tolerance against sterilizing parasites (Best *et al*., 2008, 2010) predict that genetic variation in sterilization rate is not likely to be maintained when hosts evolve resistance, because pathogens should always co-evolve to maximize sterilization rates. By contrast, tolerating infection is expected to yield genetic variation in sterilization rate (Best *et al*., 2010). Few empirical studies of tolerance have considered reproduction, but the *D. magna–P. ramosa* system offers an excellent opportunity to do so. All *Daphnia* infected with *P. ramosa* will eventually become sterilized, but some reproduction prior to sterilization is possible (Ebert *et al*., 2004; Chadwick & Little, 2005). Life-history shifts maximizing early reproduction, termed fecundity compensation, have been described in several host–parasite systems (Minchella & Loverde, 1981; Thornhill *et al*., 1986; Krist, 2001; Ebert *et al*., 2004; Chadwick & Little, 2005; Altincicek *et al*., 2008; Barribeau *et al*., 2010). In some cases, it appears that a shift in resources to early host reproduction may affect parasite densities indirectly (Ebert *et al*., 2004), but we found no effect of the amount of fecundity compensation on the total number of *P. ramosa* spores produced during the infection period (as evidenced by the lack of correlation between fecundity compensation and anti-growth resistance). Given that it results in an increase in host fitness without changing parasite densities, fecundity compensation, as we have observed it, would fit the functional definition of tolerance (Schneider & Ayres, 2008). Moreover, we identified genetic variation in the level of fecundity compensation among host genotypes. By contrast, when we analysed a second measure of fecundity tolerance – how total host fecundity (counts of offspring across their entire experimental lifetime) changed with increasing spore loads – we found that genotypes did not differ.

Tolerating the burden of infection by increasing fecundity should impact how epidemic and evolutionary dynamics proceed because the extra offspring produced are not necessarily resistant individuals. Indeed, if fecundity compensation is negatively correlated with anti-infection or anti-growth resistance, the production of extra offspring could even accelerate the epidemic by providing parasites with more susceptible hosts with low resistance to infection and/or parasite growth. Trade-offs between resistance and tolerance have been described in plants (Fineblum & Rausher, 1995; Baucom & Mauricio, 2008) and animals (Råberg *et al*., 2007) and are expected because hosts with highly efficient resistance against initial infection would be under weak selection to increase the level of tolerance, which by definition only acts once infection has established. Similarly, resistance mechanisms that fight infection by reducing parasite numbers directly would appear inconsistent with tolerance mechanisms that reduce pathogenesis without affecting parasite densities. We tested for a negative genetic correlation between the level of fecundity compensation and the two measures of resistance, but we did not detect any such trade-offs. However, an accurate measure of costs of tolerance via fecundity compensation would ideally test a much larger number of genotypes than used in the current study.

### Anti-infection resistance

Selection for anti-infection resistance has an immediate impact of reducing the prevalence of infection, and this effect is further enhanced because the presence of more resistant individuals reduces the overall risk of infection for less resistant individuals (Anderson & May, 1985). Variation at this initial stage of defence may also affect parasite evolution. If, as we observed (Fig. 1), successful establishment of infection is genetically determined, the crucial limiting step for the parasite may be gaining entry into the host, and there is no immediate advantage to parasite genotypes with higher within-host growth rates that may result in increased virulence. For this reason, in addition to lowering infection prevalence, anti-infection resistance is not predicted to affect the evolution of virulence (Gandon & Michalakis, 2000; Gandon *et al*., 2001) [although this may not always be the case (de Roode *et al*., 2011)]. However, given there is strong selection for parasite genotypes that are able to infect common host genotypes, rare host genotypes always have higher fitness than common ones. The ensuing negative frequency-dependent selection is widely acknowledged to maintain genetic variation at this stage of defence (Hamilton, 1993; Woolhouse *et al*., 2002).

### Anti-growth resistance

We observed genetic variation in parasite loads (an indication of anti-growth resistance), and such variation is probably the norm (Lambrechts *et al*., 2005, 2009; Lazzaro *et al*., 2006; Laine, 2007; Harris *et al*., 2010). In principle, differences in parasite load could arise independently of any resistance mechanism, for example, if some host genotypes offer better growth conditions for some parasite genotypes. In the case of the present experiment, by using a genetically variable parasite inoculum, and exposing hosts to wide range of infection doses, differences in parasite loads are likely to reflect a host genotype’s average ability to affect the parasite growth during infection. Genetic variation in anti-growth resistance means that in natural infection scenarios, host genotypes will vary in how many transmission-stage parasites are released during infection. Host genotypes that reduce within-host parasite growth therefore keep infection prevalence low, reducing the strength of parasite-mediated selection. However, the most resistant hosts will also select for faster-growing, potentially more virulent parasites (Gandon & Michalakis, 2000; Gandon *et al*., 2001). The optimal level of anti-growth resistance is therefore likely to vary depending on the prevalence of infection and the physiological (and by extension, evolutionary) cost of clearing infection within the host (Baalen, 1998). This interplay between the costs and benefits of anti-growth resistance across epidemiological and evolutionary time-scales could therefore explain why all hosts do not evolve to maximize the ability to clear infection.
